# A Wide-Proteome Analysis to Identify Molecular Pathways Involved in Kidney Response to High-Fat Diet in Mice

**DOI:** 10.3390/ijms23073809

**Published:** 2022-03-30

**Authors:** Elena Dozio, Elisa Maffioli, Elena Vianello, Simona Nonnis, Francesca Grassi Scalvini, Leonardo Spatola, Paola Roccabianca, Gabriella Tedeschi, Massimiliano Marco Corsi Romanelli

**Affiliations:** 1 Laboratory of Clinical Pathology, Department of Biomedical Sciences for Health, Università degli Studi di Milano, 20133 Milan, Italy; elena.dozio@unimi.it (E.D.); mmcorsi@unimi.it (M.M.C.R.); 2 Department of Veterinary Medicine and Animal Science, Università degli Studi di Milano, 26900 Lodi, Italy; elisa.maffioli@unimi.it (E.M.); simona.nonnis@unimi.it (S.N.); francesca.grassiscalvini@unimi.it (F.G.S.); paola.roccabianca@unimi.it (P.R.); gabriella.tedeschi@unimi.it (G.T.); 3 CRC “Innovation for Well-Being and Environment” (I-WE), Università degli Studi di Milano, 29133 Milan, Italy; 4 Division of Nephrology, Dialysis and Renal Transplantation, ASST Grande Ospedale Metropolitano Niguarda, 20162 Milan, Italy; leonardospatola@yahoo.it; 5 Service of Laboratory Medicine1-Clinical Pathology, IRCCS Policlinico San Donato, 20097 San Donato Milanese, Italy

**Keywords:** cardiometabolic risk, fibrosis, high-fat diet, kidney, lipids, obesity, proteomic analysis, renal damage

## Abstract

The etiopathogenesis of obesity-related chronic kidney disease (CKD) is still scarcely understood. To this aim, we assessed the effect of high-fat diet (HF) on molecular pathways leading to organ damage, steatosis, and fibrosis. Six-week-old male C57BL/6N mice were fed HF diet or normal chow for 20 weeks. Kidneys were collected for genomic, proteomic, histological studies, and lipid quantification. The main findings were as follows: (1) HF diet activated specific pathways leading to fibrosis and increased fatty acid metabolism; (2) HF diet promoted a metabolic shift of lipid metabolism from peroxisomes to mitochondria; (3) no signs of lipid accumulation and/or fibrosis were observed, histologically; (4) the early signs of kidney damage seemed to be related to changes in membrane protein expression; (5) the proto-oncogene MYC was one of the upstream transcriptional regulators of changes occurring in protein expression. These results demonstrated the potential usefulness of specific selected molecules as early markers of renal injury in HF, while histomorphological changes become visible later in obesity-related CDK. The integration of these information with data from biological fluids could help the identification of biomarkers useful for the early detection and prevention of tissue damage in clinical practice.

## 1. Introduction

Obesity is increasingly recognized as a cause of chronic kidney disease (CKD). The etiopathogenesis of obesity-related CKD is far to be unraveled fully, although different risk factors have been identified. Among these, renal lipid accumulation and lipid-induced toxicity seem to play a fundamental role in CKD emergence and progression [1]. The molecular mechanisms promoting diseases are multiple and include increased free fatty acids (FFA) uptake, overexpression of different carrier proteins (i.e., cluster of differentiation 36 (CD36)), of FFA transport proteins (i.e., SCL27A1, SLC27A2, SLC27A4), of FFA acid-binding proteins (i.e., L-FABP), impaired FFA beta oxidation, changes in the expression of genes related to lipogenesis and triglyceride hydrolysis, activation of lipid peroxidation, and production of lipo(toxic) substances [2,3,4,5,6,7,8,9]. Likely, additional unexplored molecular pathways might be involved in lipid overload-related kidney injury, such as mechanisms related to oxidative stress and inflammatory mediators.

Lipid overload and ectopic lipid accumulation in the kidney may induce inflammation and the production of reactive oxygen species (ROS), which in turn affect the structure and function of renal cells, both at the glomerular and tubular level [10], and may activate a pro-fibrotic response [4,11,12]. Renal proximal tubular cells represent the main site of lipid accumulation and are more susceptible to lipid-induced mitochondrial toxicity than other cell types due to their high-energy demand [13]. Genomic studies, both in human and animal models, have indicated that lipid deposition and fibrosis are associated with a reduced expression of mitochondrial enzymes and transcriptional factors involved in lipid oxidation as well as the increased the expression of those factors related to adipogenesis [14]. Rats fed a high-fat (HF) diet displayed chronic inflammation and high ROS levels, which resulted in the thickening of the glomerular basement membrane, extracellular matrix accumulation (ECM), and glomerulosclerosis [15].

In obesity-related CKD rats, glomerulosclerosis may also come from increased proximal tubular sodium reabsorption via sodium glucose co-transporter 1 and 2 (SGLT1 and 2), vasoconstriction of efferent arterioles secondary to Renin–Angiotensin–Aldosterone System (RAAS) activation, and hemodynamic hyperfiltration [16,17,18].

From a clinical point of view, obesity was identified as an independent predictor of new CKD onset, and it was shown to influence the prognosis of CKD due to other pathogenetic causes [19].

To date, the understanding of the pathophysiological mechanisms underlying the onset of obesity-related renal damage is still incomplete. Furthermore, we need diagnostic pathways that can identify disease onset early enough to implement interventions to prevent progression to irreversible damage.

To shed further light on the molecular pathogenesis of obesity-induced CKD, in this study, we performed quantitative proteomic and genomic assessments of kidneys of mice fed an HF diet. The specific aims of the study were: (1) to explore any potential alterations occurring in renal lipid metabolism due to HF diet, (2) to evaluate the effect of HF diet on molecular pathways leading to organ damage (i.e., fibrosis), and (3) to identify molecules that could work as potential markers of kidney damage.

## 2. Results

### 2.1. Impact of HF Diet on Lipid Metabolism-Related Transcriptome in Kidney

**Figure 1:**
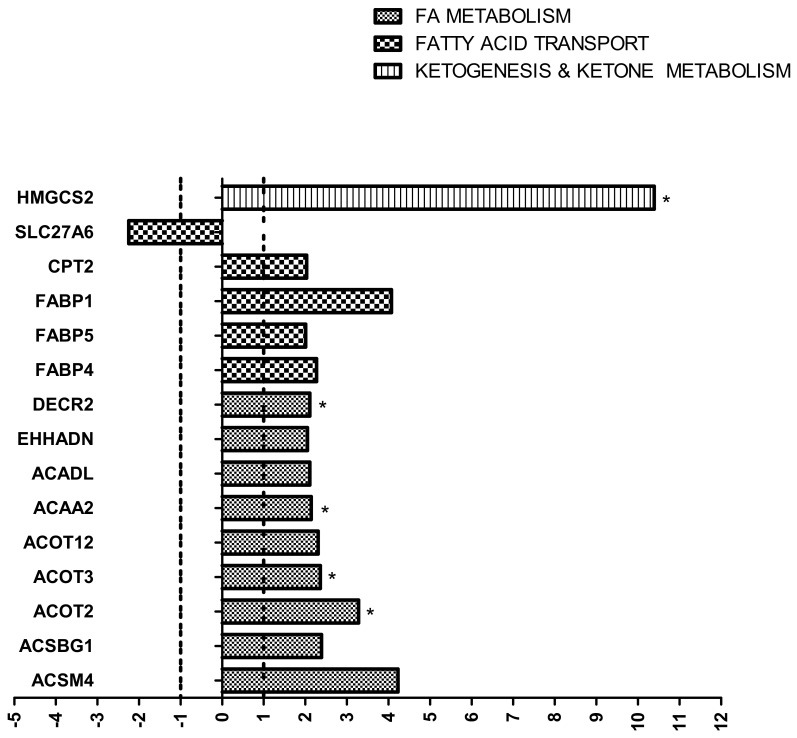
Lipid metabolism-related transcriptome in high-fat (HF) vs. normal chow (CTR) group. Gene expression profile was evaluated using the mouse Fatty Acid Metabolism RT^2^ Profiler PCR Array. Genes that showed a fold change >2 or <−2 in the HF vs. CTR group are represented and grouped according to their biological function. * *p* < 0.05.

We evaluated the expression of 84 genes involved in FFA metabolism, FFA transport, FFA biosynthesis regulation, ketogenesis and ketone body metabolism, and triacylglycerol metabolism (Supplementary File S1). Among the 84 genes, five had a cycle threshold (Ct) between 30 and 33 in both HF and normal chow (CTR) groups, which means low levels of expression. In HF kidneys, 14 genes were upregulated, and one was downregulated. Among the upregulated genes, nine were involved in FFA metabolism, four were involved in FFA transport, and one was involved in ketogenesis and ketone metabolism. Genes involved in FFA metabolism included *DECR2* (2,4-Dienoyl-CoA Reductase 2, an auxiliary enzyme of beta-oxidation), *EHHADH* (Enoyl-CoA Hydratase And 3-Hydroxyacyl CoA Dehydrogenase, one of the four enzymes of the peroxisomal beta-oxidation pathway), *ACADL* (Acyl-CoA Dehydrogenase Long Chain, an acyl-CoA dehydrogenase that catalyzes the first step of mitochondrial fatty acid beta-oxidation), *ACAA2* (Acetyl-CoA Acyltransferase 2, one of the enzymes that catalyzes the last step of the mitochondrial beta-oxidation pathway), *ACOT12*, *ACOT 3*, and *ACOT2* (Acyl-CoA Thioesterases, three enzymes that catalyze acyl-CoAs hydrolysis to the free FFA and coenzyme A, thus providing the potential to regulate intracellular levels of acyl-CoAs, free FFA, and CoASH), *ACSBG1* (Acyl-CoA Synthetase Bubblegum Family Member 1, an enzyme that catalyzes the conversion of FFA such as long-chain and very long-chain FFA to their active form acyl-CoAs for both synthesis of cellular lipids and degradation via beta-oxidation), and *ACSM4* (Acyl-CoA Synthetase Medium Chain Family Member 4, an enzyme that catalyzes the activation of FFA by CoA to produce an acyl-CoA). Genes involved in FFA transport included *CPT2* (Carnitine Palmitoyltransferase 2, a mitochondrial protein involved in uptake of long-chain FFA for their subsequent beta-oxidation in the mitochondrion), *FABP1*, *FABP5*, *FABP4* (Fatty Acid Binding Proteins, whose roles include FFA uptake, transport, and metabolism), and *HMGCS2* (3-Hydroxy-3-Methylglutaryl-CoA Synthase 2, a mitochondrial enzyme that catalyzes the first reaction of ketogenesis). The single downregulated gene was *SLC27A6* (Solute Carrier Family 27 Member 6), which is a transfer protein involved in the translocation of long-chain FFA across the plasma membrane. A statistically significant difference was also observed for *HMGCS2*, *DECR2*, *ACAA2*, *ACOT3*, and *ACOT2* (*p* < 0.05, HF vs. CTR) (Figure 1).

### 2.2. Impact of HF Diet on Fibrosis-Related Transcriptome in Kidney

We evaluated the expression of 84 genes involved in the regulation of fibrosis, which include pro- and anti-fibrotic regulators, extracellular matrix (ECM) and cell adhesion molecules, growth factors, inflammation-related molecules, signal transduction molecules, and regulators of epithelial-to-mesenchymal cell transition (Supplementary File S1). Among the 84 genes, five had a Ct between 30 and 33 in both HF and CTR groups, which means low levels of expression. In HF kidney, 17 genes were upregulated and 10 were downregulated.

In the group of genes related to epithelial-to-mesenchymal cell transition, *TGFB3* (transforming growth factor-beta, a multifunctional protein also involved in the regulation of cell adhesion and ECM formation), *SNAL1* (Snail Family Transcriptional Repressor 1, which is further involved in cell growth arrest, survival and cell migration), and *MMP3* (Matrix Metallopeptidase 3, a protein also capable of degrading several extracellular molecules and a number of bioactive molecules) were upregulated. In contrast, *ITGB1* (Integrin Subunit Beta 1, a membrane receptor which function as collagen receptor) was downregulated.

In the group of genes classified as signal transduction molecules, *CEBPB* (CCAAT Enhancer Binding Protein Beta, a transcription factor important in the regulation of genes involved in immune and inflammatory responses), *TGFB3* (already described in the previous group), and *GREM1* (Gremlin 1, DAN Family BMP Antagonist, a bone morphogenic protein antagonist with a role in tissue differentiation and fibrosis) were upregulated. Whereas, *ENG* (Endoglin, one of the major glycoprotein of the vascular endothelium exerting a critical role in the binding of endothelial cells to integrins) was downregulated. EDN1 (Endothelin, which plays a role as a vasoconstrictor peptide), which was included in the group of growth factors, was upregulated. Among the inflammatory genes, *TNF* alpha (Tumor Necrosis Factor alpha), a cytokine involved in the regulation of a wide spectrum of biological processes including cell proliferation, differentiation, apoptosis, lipid metabolism, and coagulation), *IL-5* (Interleukin 5, a cytokine that acts as a growth and differentiation factor for both B cells and eosinophils), *IL-13* (Interleukin 13, a cytokine involved in several stages of B-cell maturation/differentiation and regulation of macrophage activity, by inhibiting the production of pro-inflammatory cytokines and chemokines), *CXCR4* (C-X-C Motif Chemokine Receptor 4, a molecule endowed with potent chemotactic activity for lymphocytes), and *CCL11* (C-C Motif Chemokine Ligand 11, a protein that directly promotes the accumulation of eosinophils) were upregulated. In contrast, *IFNG* (Interferon gamma, a cytokine that triggers a cellular response to viral and microbial infections with also potential anti-fibrotic actions) and ILK (Integrin Linked Kinase, a protein with multiple cellular functions including cell migration, proliferation, and adhesion) were downregulated.

In the group of ECM remodeling genes, *TIMP2* (TIMP Metallopeptidase Inhibitor 2, an inhibitor of the matrix metalloproteinases, a group of peptidases involved in degradation of ECM), *SERPINE1* (Serpin Family E Member 1, which is the principal inhibitor of tissue plasminogen activator and inhibits the activity of matrix metalloproteinases), *SERPINE1A* (Serpin Family A Member 1, a serine protease inhibitor belonging to the serpin superfamily whose targets include elastase which cause proteolytic damage to tissues), *MMP3*, *MMP8*, and *MMP13* (Matrix Metallopeptidase 3, 8, and 13, proteins involved in the breakdown of extracellular matrix for tissue remodeling) were upregulated. In contrast, *MMP1* (Matrix Metallopeptidase 1), *TIMP3* (Tissue Inhibitor of Metalloproteinases 3, an inhibitor of MMPs), *PLG* (Plasminogen, the precursor of plasmin that dissolves fibrin of blood clots and acts as a proteolytic factor in a variety of other processes including tissue remodeling), and *PLAU* (Plasminogen Activator Urokinase, a serine protease that converts plasminogen to plasmin and is also involved in extracellular matrix degradation) are downregulated.

**Figure 2:**
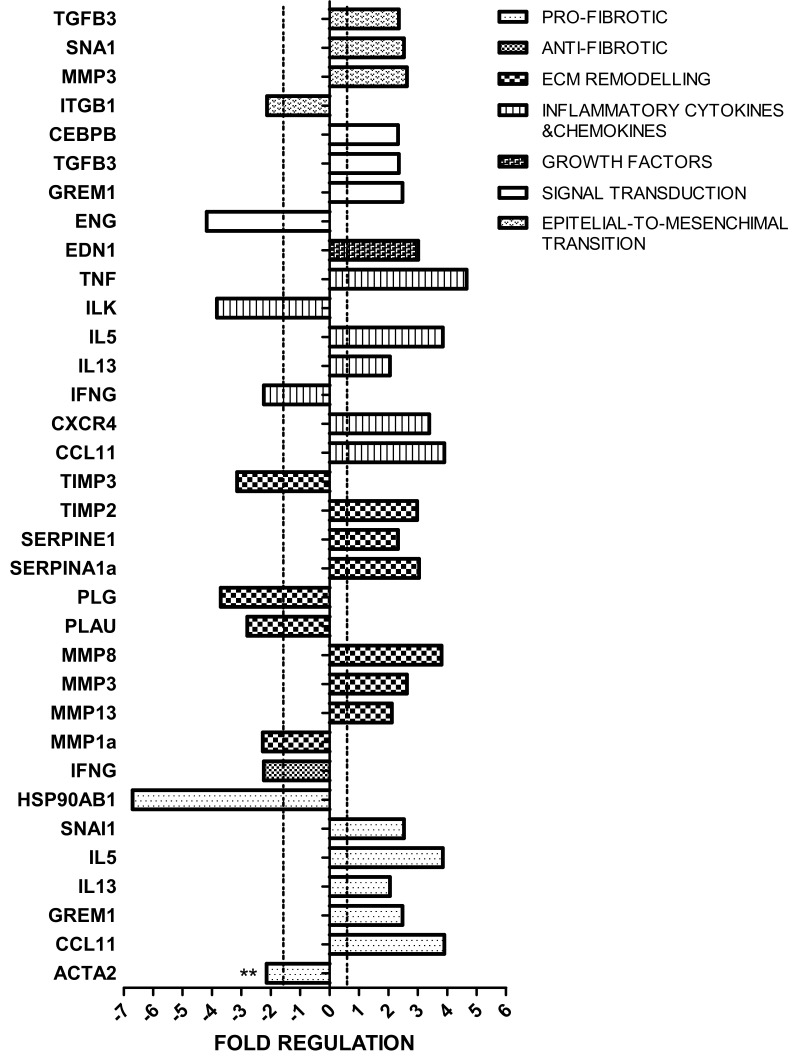
Fibrosis-related transcriptome in high-fat (HF) vs. normal chow (CTR) group. Gene expression profile was evaluated using the mouse Fibrosis RT^2^ Profiler PCR Array. Genes that showed a fold change >2 or <−2 in the HF vs. CTR group are represented and grouped according to their biological function. ** *p* < 0.01.

Among the pro-fibrotic genes, *SNAI1* (Snail Family Transcriptional Repressor 1, a transcription factor that by regulating epithelium to mesenchyme transition has been implicated in fibrosis), *IL-5*, *IL-13*, *CCL11* (inflammatory mediators with a profibrotic role), and GREM1 (already described in the signal transduction group) were upregulated. In contrast, *ACTA2* (Actin Alpha 2, a smooth muscle actin that is involved in vascular contractility, often used as a marker of myofibroblast formation) and *HSP90AB1* (Heat Shock Protein 90 Alpha Family Class B Member 1, a molecular chaperone that regulates fibroblast proliferation, myofibroblast transformation, and ECM production) were downregulated (Figure 2).

### 2.3. Impact of HF Diet on Lipid Content in Kidney

**Figure 3:**
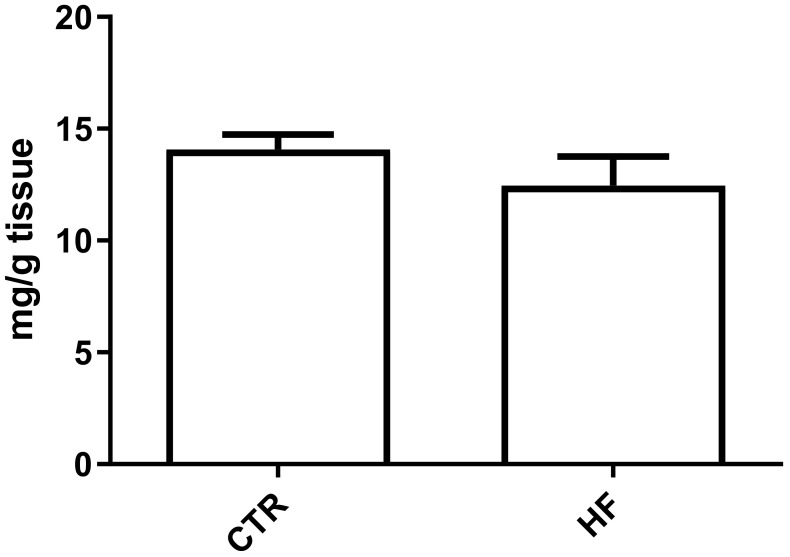
Lipid content in kidney. Quantification of neutral lipids was performed on total lipid extracts. Data are expressed as mean ± standard deviation. No difference was observed in lipid content between the high fat (HF) and normal chow (CTR) groups.

We evaluated lipid accumulation in the kidney due to HF diet. No statistically significant difference was observed between the CTR and HF groups (Figure 3).

### 2.4. Impact of HF Diet on Kidney Proteome

#### 2.4.1. Impact of HF Diet on Whole Kidney Proteome

**Figure 4:**
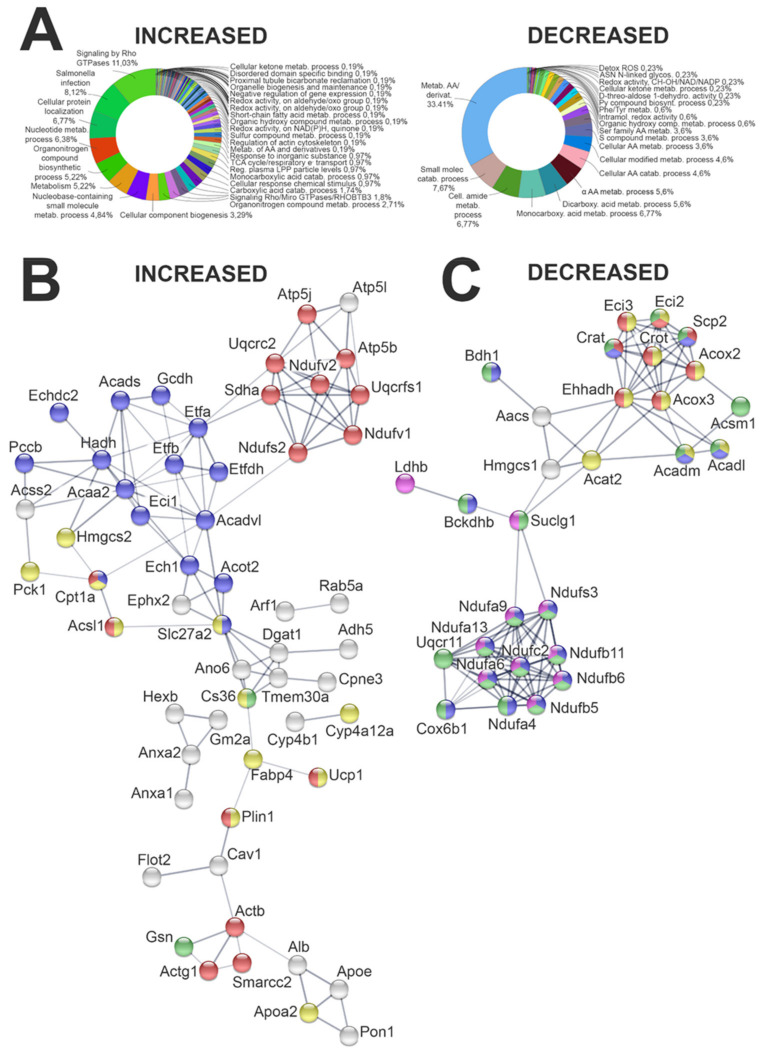
Bioinformatic analysis of the proteins increased and decreased in the comparison between the high-fat and normal chow groups. (**A**) Bioinformatic analyses by Cluego software (Cytoskape release 3.8.2) within the set of increased and decreased proteins. Functional grouping was based on *p* value ≤ 0.05. GO terms fusion allowed at least 3 counts. (**B**) Proteins increased and identified by Cluego as involved in lipid metabolism were analyzed by String (version 11.0). For clarity, the figure reports only proteins linked in a network. The color indicates proteins involved in a specific process: fatty acid catabolic process (blue), thermogenesis (red), amyloid fibril formation (green), and peroxisome proliferator-activated receptors (PPAR) signaling pathway (yellow). (**C**) Proteins decreased and identified by Cluego as involved in lipid metabolism were analyzed by String (version 11.0). For clarity, the figure reports only proteins linked in a network. The color indicates proteins involved in a specific process: peroxisome (red), mitochondria (green), mitochondrial envelop (blue), fatty acid beta oxidation (yellow), tricarboxylic acid cycle (TCA), and electron transport (magenta).

To evaluate the impact of HF diet on kidney proteome, a quantitative shotgun label-free strategy was used to compare the entire kidney proteome of the CTR and HF group. The analysis identified 1795 proteins in the CTR group and 1913 in the HF group. A total of 1727 proteins was found to be expressed in both groups, of which some were more or less expressed in HF than the CTR group. By including proteins exclusively expressed either in HF (186) or in CTR (68), 589 proteins were found to be increased and 340 decreased in the HF vs. CTR group (Supplementary Files S2 and S3). The bioinformatic analysis, carried out on these proteins by Cluego and Panther software, clearly demonstrated a great impact of the HF diet on the whole kidney proteome. Endocytosis and vesicle mediated transport, protein localization, actin cytoskeleton organization, regulation of cell shape, cytoskeletal regulation by Rho GTPase, and integrin signaling are strongly enriched among the increased proteins in the HF group, as well as many proteins involved in lipid metabolism, thermogenesis, mitochondrial energetic metabolism, and PPAR (peroxisome proliferator-activated receptor) signaling (Supplementary Files S4 and S5, Figure 4A Increased).

The impact of HF diet on metabolism was even more striking in the decreased data set where more than 33% of the decreased proteins were related to amino acid metabolism, TCA (tricarboxylic acid) cycle, PPAR signaling, peroxisomal lipid metabolism, and cellular response to stress (Supplementary Files S4 and S5, Figure 4A Decreased).

**Figure 5:**
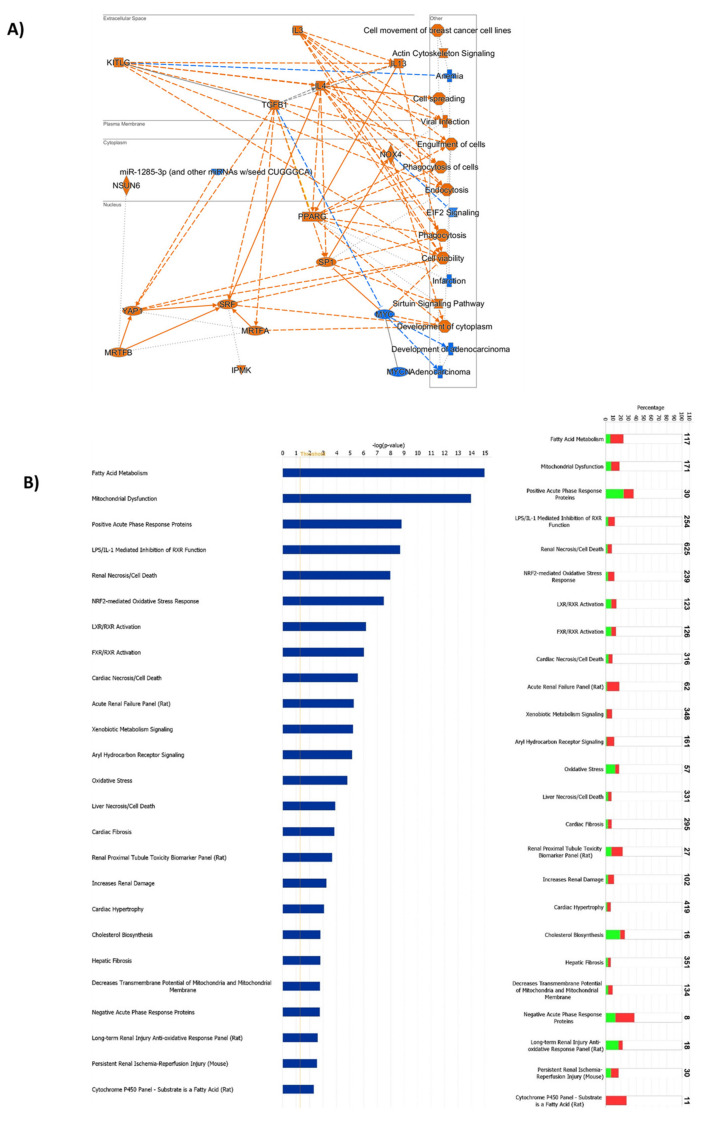
Bioinformatic analysis by Ingenuity Pathway Analysis (IPA) of the proteins differentially expressed in the comparisons between the high-fat and normal chow group. (**A**) Graphical summary of the bioinformatic analysis. Increased proteins/processes are indicated in orange and decreased proteins/processes are indicated in blue. (**B**) Toxicity list. For clarity, only the top 25 are indicated in the histograms. The histogram on the left reports the log *p*-value, and the significance threshold is indicated with the orange lane. The histogram on the right indicates the percentage of genes in our data set that are present in a specific cohort whose composition is indicated on top of each bar. Color code: red = upregulated proteins, green = downregulated proteins, white = no overlap with the data set.

The analysis by Cluego and Panther software was confirmed by the Ingenuity Pathway Analysis (IPA), whose results are graphically summarized in Figure 5A and in Supplementary File S6. The top five most enriched pathways in the analysis were: remodeling of epithelial adherens junctions (increased), EIF2 (Eukaryotic Initiation Factor 2) signaling (decreased), sirtuin signaling pathway (increased), mitochondrial dysfunction, and oxidative phosphorylation (increased). These results were in accordance with the analyses performed by CLUEGO and Panther software.

IPA allowed the identification of the cascade of upstream transcriptional regulators that can explain the changes occurring in protein expression. Interestingly, one of the upstream regulators identified was the proto-oncogene MYC. As expected, renal damage, tubule and glomerular injury, and nephrosis are indicated among the top diseases that can fit with the changes of protein expression observed in the HF group. Fatty acid metabolism and mitochondrial dysfunction were the most affected pathways (Figure 5B and Supplementary File S6).

#### 2.4.2. Evaluation of Lipid Metabolism-Related Proteome in Kidney

The analysis of the whole kidney proteome strongly suggested a significant alteration in the expression of proteins involved in lipid metabolism. Specifically, the bioinformatics analysis performed by Cluego software indicated that 96 molecules were increased and 37 were decreased in the HF vs. CTR group. These proteins were analyzed by the String software to better describe protein–protein interactions and enrichment of functional networks. The analysis showed that most of these proteins are linked together in a very large network, as summarized in Figure 4B (increased proteins) and Figure 4C (decreased proteins). Among the 96 increased proteins, more than more than one-third were mitochondrial proteins, and many of them were related to endoplasmic reticulum, plasma membrane, extracellular matrix, and vesicles. Lipid transport, storage, and localization were increased in the HF group (Supplementary File S4 and Figure 4). In the decreased data set, 31 proteins out of 37 were membrane-related proteins. Among these, 17 were in the mitochondrial membrane (Supplementary File S4).

Lipid catabolism was also affected: 24 proteins were increased in the HF group, while 13 related to thermogenesis and peroxisomal FFA beta-oxidation by acyl-CoA oxidase were decreased. Many peroxisomal proteins and components of the PPAR signaling pathway were also altered. Accordingly, FFA beta oxidation using acyl-CoA dehydrogenase, short-chain fatty acid metabolic processes, TCA cycle, and oxidative phosphorylation were increased, as well as activation of BAD (BCL2 associated agonist of cell death) and translocation to mitochondria. On the contrary, enzymes involved in amino acids catabolism were decreased (Supplementary File S4 and Figure 4B,C).

**Figure 6:**
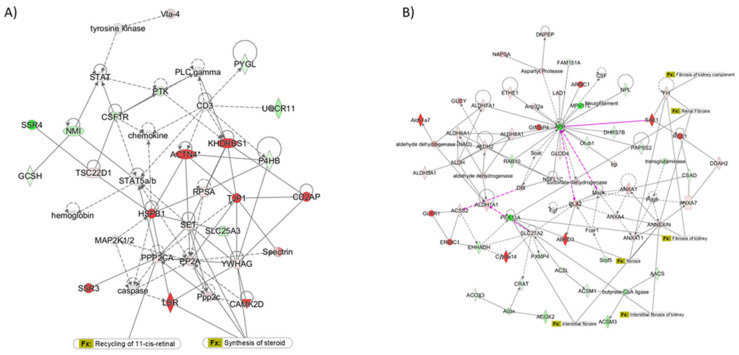
Graphical representation of networks identified by Ingenuity Pathway Analysis (IPA). (**A**) The network of lipid metabolism was merged with energy production and endocrine system disorders. Increased proteins are indicated in red and decreased proteins are indicated in green. (**B**) Graphical representation of the network of glomerular injury. Increased proteins are indicated in red and decreased proteins are indicated in green.

Networks related to metabolic diseases and lipid metabolism were the most enriched in the IPA analysis, as shown in Figure 6A, which indicates that the proteins altered by HF diet in the network of lipid metabolism merged with energy production and endocrine system disorders. Out of the 84 genes involved in steatosis analyzed by transcriptomics (Figure 1), proteomic analysis confirmed HMGCS2, CPT2, FABP4, ACAA2, ACOT2, CPT1A, and ALDH2 among the upregulated proteins and BDH1 and ACADL in the decreased data set.

#### 2.4.3. Evaluation of Fibrosis-Related Proteome in Kidney

The bioinformatic analysis carried out on the proteins differentially expressed in the two groups highlighted many changes in adhesion molecules, growth factors, extracellular matrix, and cytoskeletal components, which may be easily linked to obesity-related renal damage. IPA analysis suggested that some alterations occurring in the kidney proteome of the HF group are indicative of both tubular and glomerular injury. Namely, 29 molecules were suggestive of tubule injury and 35 molecules were suggestive of glomerular injury (Supplementary File S6).

A graphical visualization of the network of proteins related to glomerular injury is reported in Figure 6A. The links to lipid metabolism were also indicated to stress the close relationship between lipid metabolism and renal fibrosis (PPP2CA, PP2A, YWHAG, LBR).

Similarly, in the lipid network, PEX11A, ANNEXIN, ANXA7, and FH were also linked to kidney fibrosis (Figure 6B). Despite being downregulated in transcriptomics, ITGB1 and the integrin signaling pathway were increased at the proteomic level (Supplementary Files S4 and S5). In addition, two proteins involved in lipid metabolism, namely CD36 and FLOT2, were increased (Figure 4B).

Other proteins possibly involved in renal fibrosis were indicated in the IPA disease annotation and toxicity lists, which showed genes known to be involved in a particular disease. The results were reported in the Supplementary File S6. APRT, ATP1B1, C3, CAV1, FH, ITGB1, LGMN, MAN2A1, PEX11A, and SLC22A6 were indicated as markers of renal fibrosis and HNMT, Cyp4a14, ACSM3 were in the panel of biomarkers for reversible glomerulonephritis in rat. Instead, ACE, APOE, C3, FN1, LCN2, SIGIRR, SLC22A1, and SLC22A2 were markers of renal tubule damage, and HSPB1, SLC25A11, SLC27A2, SLC22A6, SLC22A2, GSTP1 were listed in the panel of biomarkers for renal proximal tubule toxicity.

### 2.5. Evaluation of Kidney Morphology, Fibrosis, Amyloidosis, and Other Kidney Lesions

#### 2.5.1. Hematoxylin–Eosin Staining

**Figure 7:**
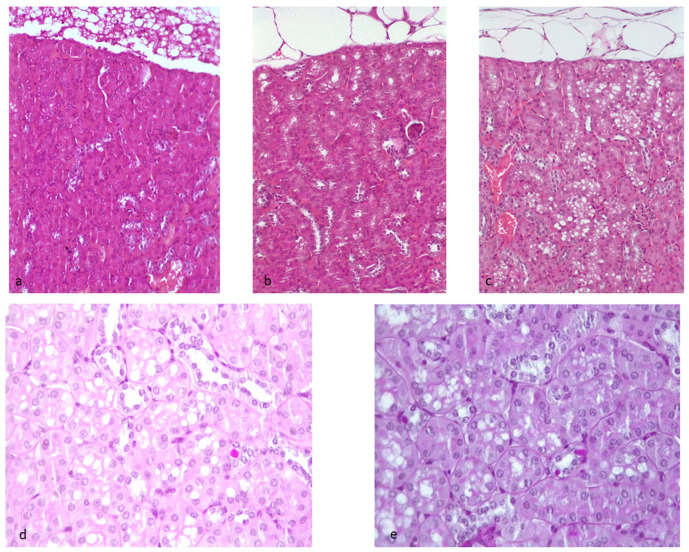
Histological examination. Hematoxylin- and eosin-stained kidney sections from normal chow (CTR) or high-fat (HF) fed mice at the end of 20 weeks. (**a**) CTR, normal cortex; (**b**) HF mouse: tubular vacuolation score 1, (**c**) HF mouse: tubular vacuolation score 2. PAS-stained kidney sections from HF-fed mice at the end of 20 weeks. (**d**) HF-mouse score 1: one glycogenated nucleus is visible; (**e**) HF mouse score 2: three glycogenated nuclei are visible.

Hematoxylin–eosin staining was applied to renal tissue sections to assess the overall morphology. Prominent (significant) vacuolization of the tubular epithelial cells of proximal convoluted tubules was evident in the HF group. These vacuoles did not stain with PAS, Masson’s Trichrome, nor Congo red. The only background lesion (“normal”) in relation to age at the endpoint was a minimal to moderate (only in one) inflammatory infiltration by small mature lymphocytes and rare mature plasma cells at the level of the connective tissue of the pelvis (subepithelial) (Figure 7 and Supplementary File S7, Table S1).

#### 2.5.2. Masson’s Trichrome Staining

Masson’s trichrome staining was applied to renal tissue sections to assess fibrosis. No differences in glomerular or tubulointerstitial fibrosis in the HF group were detected. In some mice (consistent with age of study endpoint), mild tubulointerstitial fibrosis was observed (Figure 7 and Supplementary File S7, Table S2).

#### 2.5.3. Congo Red

Congo red stain was consistently negative excluding amyloid deposition in the kidneys of all mice.

#### 2.5.4. PAS

PAS was used to examine and score tubular lesions. Assessment confirmed a significant increase in proximal tubular cell vacuolation in HF. Vacuoles were consistently PAS negative. PAS demonstrated an increase (not significant) of glycogenated nuclei in the HF group (Figure 7 and Supplementary File S7, Tables S2 and S3). After counting, the median (25°–75° percentiles) was 4 (1–7) in the HF group and 1 (0–2.5) in the CTR group. We found no significant difference between the two groups (*p* = 0.175), but a slight shift was observed (more positive nuclei in the HF group) (Supplementary File S7, Tables S2 and S3).

## 3. Discussion

We evaluated the effect of HF diet on kidney by integrating genomic, proteomic, and histological analyses with the final aim of identifying potential markers of kidney diseases in 26-week-old mice fed for 20 weeks an HF diet, with particular interest in ectopic lipid accumulation and tissue fibrosis. The main findings can be summarized as follows: (1) HF diet induced the activation of specific pathways leading to fibrosis and increasing fatty acid metabolism; (2) HF diet promoted a metabolic shift of the lipid metabolism from the peroxisome to the mitochondria; (3) no signs of glomerular fibrosis were observed histologically; (4) the early signs of kidney damage seemed to be related to changes in membrane protein expression and/or oxidative stress; (5) the proto-oncogene MYC was one of the upstream transcriptional regulators that can explain the changes occurring in protein expression.

### 3.1. Lipid Metabolism

Gene expression analysis suggested a general activation of genes involved in lipid oxidation. Out of 84 genes involved in lipid metabolism analyzed by transcriptomics, proteomic analysis confirmed HMGCS2, CPT2, FABP4, ACAA2, ACOT2, CPT1A, and ALDH2 among the upregulated proteins, while BDH1 and ACADL were in the decreased data set. CPT1A and CPT2 are mitochondrial proteins important for lipid translocation through the mitochondrial membrane, FABP4 is a fatty acid-binding protein, and ACAA2 and ACOT2 are involved in lipid beta-oxidation. ACADL, whose transcript increased in HF, is listed among the proteins decreased in accordance with a general decrease in the peroxisomal activity that oxidized mainly long-chain and branched FFA and is in line with the downregulation of SLC27A6 observed by transcriptomics. The changes occurring due to HF diet indicated an overall increase in lipid transport, storage, and localization (6, 12, and 29 proteins, respectively), and a shift of lipid metabolism from the peroxisome to the mitochondria, mainly due to the downregulation of many peroxisomal proteins and components of the PPAR pathway [20]. At the mitochondrial level, the beta-oxidation of lipids was the main fuel of energy generation, as confirmed by the decreased expression of enzymes involved in the amino acid catabolism.

Peroxisomes perform both catabolic as well as anabolic functions. The role of peroxisomes in metabolism is strongly related to the interaction with other subcellular organelles, mainly mitochondria. In fact, the beta-oxidation of FFA in peroxisomes produces chain-shortened acyl-CoAs, which can only be fully oxidized in mitochondria. Furthermore, the reoxidation of NADH (nicotinamide adenine dinucleotide) produced in peroxisomes back to NAD+ is performed in mitochondria. As with mitochondria, peroxisomes contain an FFA beta-oxidation machinery. Although beta-oxidation in peroxisomes and mitochondria are basically identical, there are major differences between the two systems: (1) enzymes involved in the four steps of beta oxidation are different and are encoded by different genes; (2) enzymes catalyzing the first step of beta-oxidation are FAD (flavin adenine dinucleotide)-dependent dehydrogenases in mitochondria and FAD-dependent acyl-CoA oxidases in peroxisomes, with the first transferring electrons to the Electron-Transfer-Flavoprotein cycle and the second transferring electrons directly to molecular oxygen; (3) FFA cross the mitochondrial membranes as acylcarnitine esters through the action of CPT1, CPT2, and CACT, whereas they are transported across the peroxisomal membrane as acyl-CoAs, or as free FFA; (4) peroxisomes can only chain-shorten FFA to acetyl-CoA, propionyl-CoA, and different medium-chain acyl-CoAs, whereas in mitochondria, FFA are totally metabolized to CO_2_ and H_2_O [21].

Previous studies suggested that defects in peroxisome function could result in lipid accumulation and induce mitochondrial dysfunction by promoting mitochondrial depolarization, respiratory chain dysfunction, oxidative stress, vacuolization, and/or the release of cytochrome c in association with cell death [22,23,24,25]. In our study, we did not observe lipid accumulation. Both the lipid quantification and the histological analyses seemed to confirm the absence of steatosis in the HF group. The main microscopical findings included proximal tubular vacuolization and glycogenated nuclei in HF mice. Proximal tubular vacuolation was the single statistically significant morphological change and represented the single morphological lesion that could be considered an early renal change in obesity-related CKD [26]. However, this lesion could not be clearly interpreted morphologically, as the specific content of the vacuoles was not clearly demonstrated because the vacuoles were negative for all the stainings utilized. Unfortunately, tissue processing extracts lipids from tissues, thus hampering their identification by the corresponding specific stains. Despite the speculation on the possible accumulation of lipids stored in the proximal tubule vacuoles, this hypothesis is contrasted by the finding of the lack of a statistically significant lipid accumulation difference between mice fed normal chow and an HF diet. Furthermore, lymphocyte infiltrate, a key factor in tissue inflammation associated with steatosis, was not observed [1]. It is possible to speculate that this early morphological lesion may derive from membrane dysfunction and excessive water accumulation that could be related to the observation of increased expression of endocytosis and vesicle-mediated transport, protein localization, actin cytoskeleton organization, regulation of cell shape, and cytoskeletal regulation by Rho GTPase, leading to altered water-pump and membrane dysfunction.

A previous study by Decleves et al. [26] observed in C57BL/6J mice fed a 14-week HF diet proximal tubular injuries with enlarged vacuoles. The vacuoles were almost composed of cholesteryl esters and phospholipids, whereas triglyceride levels were unchanged between groups, as well as long-chain and short chain non-esterified FFA. Other studies performed on the same animal model showed marked glomerular and tubular injuries, an increased content of triglycerides and cholesterol, activated lipogenic pathways for cholesterol and triglyceride synthesis, increased oxidative stress, tubular cell death, an increased accumulation of type IV collagen in glomeruli, and an increase in macrophage infiltration in the renal medulla [27,28]. Since our lipid quantification was performed by a quantification kit that specifically measures neutral lipids, we cannot exclude the accumulation of phospholipids, at least. Despite the increased lipid availability, we could observe only early signs of lesions. We know that the characteristics of obesity induced by an HF diet are influenced by the sex, strain, and age of mice. Looking for a potential explanation of the differences observed between ours and the previous studies, we can just consider that our mice were fed for a longer time (20 weeks vs. 16) and were 6 weeks older at sacrifice. We cannot exclude the activation in our model of some compensatory mechanisms, such as hepatic lipid metabolism and systemic metabolism [29]. Maybe, the upregulation of proteins involved in mitochondrial beta-oxidation worked as a preventive measure just against lipid deposition. Two additional considerations must be completed: (1) the marked activation of mitochondrial beta-oxidation can be a risk factor for the onset of organ damage, especially due to the increased production of ROS [30], and (2) the decreased peroxisome metabolism induced by an HF diet can lead to long-term mitochondrial disfunctions [31]. Proteomic data confirmed, in fact, that 17 out of the 31 decreased membrane proteins were located just in the mitochondrial membranes. Our data did not prove a causal relationship between peroxisome and mitochondrial dysfunctions, and we did not measure ROS, but given that peroxisomes play a central role in lipid metabolism and oxidative stress, it is likely that changes occurring in mitochondrial membrane composition and mitochondrial function can be indirectly promoted by peroxisome disfunction [24].

Certainly, further analyses will have to be completed at the level of metabolic functionality to completely understand and characterize changes occurring in lipid metabolism and the related consequences.

### 3.2. Fibrosis

Gene expression study indicated the activation of both pro-fibrotic and anti-fibrotic pathways, with a general imbalance in favor of pro-fibrotic. However, morphological analyses identified only a moderate fibrosis that was age-related and not significantly associated with HF diet. The increase, although not significant, of the vacuolated nuclei suggested the onset of a damage, but since it was not directly attributable to the fibrotic process, it could indicate possible tubular insults of other origins. Overall, although of less significance, we can confirm that most of the changes observed mainly affected the tubules.

The integration of genomic with proteomic data highlighted the activation of tissue remodeling and the loss of cell–extracellular matrix interaction. IPA analysis confirmed the activation of pathways associated with remodeling and fibrosis in addition to FFA acid oxidation and mitochondrial dysfunction. Alteration of the c-Myc proto-oncogene was one of the upstream regulators of these modifications. c-Myc encodes a pleiotropic transcription factor that participates in the regulation of various genes, including genes vital for regulating the cell cycle, cell proliferation, and apoptosis. The overexpression of c-Myc was identified as a stimulus of proliferation and activation of renal fibroblasts, and targeting c-Myc was shown to be of clinical utility in the treatment of renal fibrosis [32]. The involvement of c-Myc in fibrosis was also shown in other organs, such as the liver and lung [33,34]. The downregulation of c-Myc observed in our model could explain the absence of obvious signs of fibrosis. Relevant to this study, in fact, was the lack of morphological changes related to early fibrosis, contrasted with the increased expression of ECM remodeling and profibrotic molecular pathways/markers in HF kidneys. Although other studies observed an increased accumulation of type IV collagen in glomeruli at earlier time-points and/or glomerular injury by electron microscopy [27,28], we could not confirm these observations and, as previously discussed for steatosis, we cannot exclude the activation in our model of early mechanisms that protect against fibrosis. Of interest is the upregulation of proteins involved in autophagy, which is the natural degradation of damaged organelles, cell membranes, and proteins through a lysosome-dependent regulated mechanism. Autophagy activation appeared to elicit protective functions in in vivo models of pulmonary fibrosis [35], presumably by enhancing the resistance of alveolar epithelial cells to programmed death, reducing TGFB-dependent fibroblast differentiation, and suppressing the inflammatory cascade. Overall, these observations can suggest that autophagy in the HF kidney may be functional to counteract the fibrosis. These results demonstrate the usefulness of specific selected molecular markers as early indicators of chronic renal injury in HF, while histomorphological changes of glomerular and tubulointerstitial fibrosis become visible late in CDK progression. Therefore, these pathways can represent early indicators of damage and could allow for early diagnosis and therapy.

## 4. Materials and Methods

### 4.1. Animal Model

Fourteen six-week-old male C57BL/6N mice (Charles River Laboratories, Calco, Italy) were divided into two groups and fed for 20 weeks as follows: (1) normal chow diet (10% fat, CTR) and (2) high-fat (HF) diet (60% fat) (Charles River Laboratories). The mice were housed at constant room temperature (22 ± 2 °C) and humidity (60 ± 5%) with a light–dark cycle of 12 h each with water ad libitum. At the age of 26 weeks, the mice were sacrificed through exposure to atmosphere saturation of carbon dioxide for 15 min. Kidneys were collected, immediately snap-frozen in liquid nitrogen, and stored at −80° until analyses or fixed in 10% buffered formalin, routinely processed, and paraffin-embedded. The Italian Ministry of Health approved all animal procedures (Number 5AD83.N.G1Q).

### 4.2. Total RNA Extraction and Reverse Transcription

Disruption and homogenization of kidney samples (*n* = 6 for each experimental group) were performed with the TissueLyser II equipment (QIAGEN, Milan, Italy) through high-speed shaking in plastic tubes with stainless steel beads. Then, total RNA was isolated using the RNeasy Lipid Tissue Mini Kit (QIAGEN), according to the manufacturer’s instructions. RNA concentration was quantified with NanoDrop (Thermo Fisher Scientific, Monza, Milan, Italy). RNA samples (1 μg) were first treated with a genomic DNA elimination step (5 min/42 °C and kept on ice at least 1 min) and then reversely transcribed using the RT^2^ First Strand Kit (15 min/42 °C and 5 min/95 °C) (QIAGEN). Samples were stored at −20 °C until real-time polymerase chain reaction (qPCR) analysis.

### 4.3. RT^2^ Profiler PCR Arrays

RT^2^ Profiler PCR Arrays allowed the detection of 84 key gene transcripts related to mouse fatty acid metabolism (PAMM-007Z, QIAGEN) and 84 key gene transcripts related to mouse fibrosis (PAMM-120Z, QIAGEN) using qPCR. Each cDNA sample was diluted with nuclease-free water and mixed with the RT^2^ SYBR green Mastermix (QIAGEN). Twenty-five μL of the same experimental mixture were automatically added to each well of the array (one array for each cDNA) using the QIAgility^®^ equipment (Qiagen). qPCR was performed by the RotorGene-Q (Qiagen) and consisted of an initial activation of the Hotstart DNA Taq polymerase at 95 °C/10 min, which was followed by 40 cycles of 95 °C/15 s and 60 °C/30 s. Then, dissociation curves were performed to verify the specificity of the amplicons using the default melting curve program of the instrument. Data were analyzed using the RT2 Profiler PCR Array Data Analysis Web Portal (QIAGEN). A list of all the transcripts included in the fatty acid metabolism-array and in the fibrosis-array is included in Supplementary File S1.

### 4.4. Analysis of RT2 Profiler PCR Arrays

Each array includes 5 housekeeping genes for normalization, 1 genomic DNA control, 3 reverse transcription controls, and 3 positive PCR controls. The same threshold was applied to all the fatty acid metabolism arrays and the fibrosis arrays for data analysis. A Ct >35 was considered a negative call. Ct for genomic DNA controls >35 and Ct of 20 ± 2 for the positive PCR controls confirmed the lack of DNA contamination and efficient PCR amplification, respectively. Normalization of data expression can be performed using one of the housekeeping genes or any other of the 84 genes at the condition that the Ct value of the gene used for normalization does not differ by more than 1.5 cycle across arrays. Then, normalization was performed by calculating the ΔCt for each gene in the plate. The RT^2^ Profiler PCR Array Data Analysis Web Portal was used to calculate the fold change based on the ΔΔCt method. For fold-change values greater than 1, the results were reported as fold upregulation. For fold-change values less than 1, the negative inverse of the results was reported as fold downregulation. Genes that showed a fold change >2 in the HF vs. CTR group were shown. The calculated *p*-values were based on Student’s *t*-test (two-tail distribution and equal variances between the two samples) on the replicate 2-ΔCt values for each gene in the HF compared to the CTR group and considered significant for *p*-values < 0.05 [36].

### 4.5. Lipid Extraction and Quantification

Total lipids were extracted from tissues using the Lipid Extraction Kit from cell Biolabs (#STA-612 San Diego, CA, USA). Tissues (*n* = 6 for each experimental group) were homogenized in phosphate-buffered saline solution (PBS) at the final concentration of 2 mg/mL. One hundred µL of whole tissue homogenates were added with reagents provided in the kit and centrifuged according to the manufacturer’s instructions to obtain a final organic phase containing the lipids. Samples were dried down at 37 °C and then resuspended in chloroform. Then, quantification was performed in a 96-well plate by the fluorimetric lipid quantification kit from Biolabs (#STA-617) according to the instructions manual. Standards and samples were read at 450 nm excitation and 585 nm emission with the GloMax^®^-Multi Microplate Multimode Reader (Promega, Milan, Italy).

### 4.6. Shotgun Mass Spectrometry Analysis for Label-Free Proteomics

Kidney tissues of 8 CTR mice and 8 mice fed an HF diet were analyzed by a shotgun label-free proteomic approach for the identification and quantification of expressed proteins. The tissues were homogenized in 100 μL of extraction buffer (8 M urea, 20 mM Hepes pH 8.0, with Protease and Phosphatase Inhibitors cocktail) as previously reported [37]. Prior to proteolysis, proteins were reduced with 13 mM dithioerythriol (30 min at 55 °C) and alkylated with 26 mM iodoacetamide (30 min at room temperature). Protein digestion was performed using sequence-grade trypsin (Promega) for 16 h/37 °C using a protein/enzyme ratio of 20:1 [38]. The collected peptides were desalted using Zip-Tip C18 before Mass Spectrometric (MS) analysis. NanoHPLC coupled to MS/MS analysis was performed on a Dionex UltiMate 3000 directly connected to an Orbitrap Fusion Tribrid mass spectrometer (Thermo Fisher Scientific) by a nanoelectrospray ion source. Peptide mixtures were enriched on 75 μm ID × 150 mm EASY-Spray PepMap RSLC C18 column (Thermo Fisher Scientific) and separated using the LC gradient: 1% ACN in 0.1% formic acid for 10 min, 1–4% ACN in 0.1% formic acid for 6 min, 4–30% ACN in 0.1% formic acid for 147 min, and 30–50% ACN in 0.1% formic for 3 min at a flow rate of 0.3 μL/min. Orbitrap-MS spectra of eluting peptides were collected over an *m/z* range of 375–1500 at resolution of 120,000, operating in a data-dependent mode with a cycle time of 3 s between master scans. HCD MS/MS spectra were acquired in Orbitrap at resolution of 15,000 using a normalized collision energy of 35% and an isolation window of 1.6 *m/z*. Dynamic exclusion was set to 60 s. Rejection of +1 and unassigned charge states were enabled [39]. A database search was conducted against the Mus Musculus Uniprot sequence database (https://www.uniprot.org/proteomes, accessed on 4 March 2019) with MaxQuant (version 1.6.0.1) software. The initial maximum allowed mass deviation was set to 10 ppm for monoisotopic precursor ions and 0.5 Da for MS/MS peaks. Enzyme specificity was set to trypsin, defined as C-terminal to Arg and Lys excluding Pro, and a maximum of two missed cleavages were allowed. Carbamidomethylcysteine was set as a fixed modification, while N-terminal acetylation, Met oxidation, and Asn/Gln deamidation were set as variable modifications. Quantification in MaxQuant was performed using the built-in label-free quantification algorithms (LFQ) based on extracted ion intensity of precursor ions. False protein identifications (1%) were estimated by searching MS/MS spectra against the corresponding reversed-sequence (decoy) database [40].

Statistical analysis was performed using the Perseus software (version 1.6.14.0). Only proteins that were present and quantified in at least 75% of the repeats were positively identified in a sample and used for statistical analysis. Proteins were considered differentially expressed if they were present only in one condition or showed a significant *t*-test difference (*p* ≤ 0.05) [41].

Bioinformatic analyses were carried out by Panther software (release 16.0) [42], CLUEGO software (Cytoskape release 3.8.2) [43], and Ingenuity Pathway Analysis (IPA 2021 release) to cluster enriched annotation groups of biological processes, pathways, and networks within the set of identified proteins. Functional grouping was based on Fischer’s exact test *p*-value ≤ 0.05 and at least 3 counts.

The mass spectrometry proteomics data have been deposited to the ProteomeXchange Consortium via the PRIDE [44] (PXD031915)

### 4.7. Tissue Preparation, Histological Examination, and Scoring

Five animals per group were examined. Formalin-fixed and routinely processed hemisected kidneys were embedded in paraffin, 4-micron sections were cut and stained with hematoxylin and eosin, Massons Thricrome, Congo red, and Periodic Acid Schiff (PAS). One ECVP board certified anatomical pathologist performed histological analyses using a light microscope (Leika DM1000 photomicroscope linked to a ICC50W Leika digital camera). Lesion were assessed and scored as previously described by Glastras et al. [45] with modifications for parameters examined at 400× magnification (tubular lesions) by standardizing the area of assessment to 2.37 mm^2^ [46]. One kidney hemisection was examined at 100× magnification for foci of tubulointerstitial fibrosis and graded using a scale of 0 to 4 (0—normal; 1—involvement of <10% of the cortex; 2—involvement of 10–25% of the cortex; 3—involvement of 25–75% of the cortex; and 4—extensive damage involving >75% of the cortex).

For glomerulosclerosis, the first 20 randomly selected glomeruli at the kidney cortex were examined and graded with Masson’s trichrome stain and PAS. The sections were scored as 0—normal, 1—<25% involvement, 2—<50% involvement, 3—<75%, and 4—>75% sclerosis, and then, the average of 20 individual scores was calculated to generate the glomerulosclerosis score. Tubular lesions were scored using a 2.37 mm^2^ area by selecting 12 random non-overlapping high-power fields (HPFs) of renal cortex at 400× magnification with a microscope characterized by 10× ocular objective with FN20 mm [46], including (A) tubular vacuolation, (B) tubular dilatation, (C) glycogenated nuclei, and (D) tubular cast. For tubular vacuolation, the following scores were used: 0, absence; 1, <25% of the cortical tubules have vacuoles; 2, 25–50% of the cortical tubules have vacuoles; 3, >50% of cortical tubules have vacuoles. For tubular dilatation, the following scores were used: 0, absence; 1, <5 dilated cortical tubules were observed per HPF; 2, 5–10 dilated cortical tubules were observer per HPF; 3, >10 dilated tubules were observed per HPF. For glycogenated nuclei in tubular epithelium, the following scores were used: 0, absence; 1, only 1 glycogenated nuclei observed per HPF; 2, 2 to 3 glycogenated nuclei observed per HPF, 3, >3 glycogenated nuclei observed per HPF. For tubular casts, the following scores were used: 0, absence; 1, present.

### 4.8. Statistical Analysis

Analysis of genomic and proteomic data can be found in Section 2.4, respectively. Concerning other analyses, data are expressed as mean ± SD or median and 25°–75° percentiles. The Kolmogorov–Smirnoff test was used to evaluate the normality of data distribution. Group comparisons were performed by t-test or Mann–Whitney tests, as appropriate. The GraphPad Prism 5.0 biochemical statistical package (GraphPad Software, San Diego, CA, USA) was used for data analyses. A *p*-value < 0.05 was considered significant.

## 5. Conclusions

The HF diet is known to promote kidney insults that can further progress to CKD. Although previous data obtained in the same animal model indicated signs of kidney steatosis and fibrosis, we observed just early signs of damage, despite the activation and modulation of many different pathways related to lipid metabolism, fibrosis, and oxidative stress. The proteomic approach allowed us to identify differentially expressed molecules and pathways that can work as potential early signs of disease. The translation of these data in the clinical practice needs further investigation. In fact, most of the molecules identified were intracellular components that are not expected to be detectable in plasma or other biological samples such as urine. In addition, the integration of these data with data from biological fluids could help the identification of targets of interest for the clinical practice.

## Data Availability

The data used to support the findings of this study are available from the corresponding author upon request. The mass spectrometry proteomics data have been deposited to the ProteomeXchange Consortium via the PRIDE [44] partner repository, with the dataset identifier PXD031915.

## Supplementary Materials

The following supporting information can be downloaded at: https://www.mdpi.com/article/10.3390/ijms23073809/s1.
