# Supplementary material for: A Wide-Proteome Analysis to Identify Molecular Pathways Involved in Kidney Response to High-Fat Diet in Mice
Source: Int J Mol Sci. 2022 Mar 30;23(7):3809. doi: 10.3390/ijms23073809 (PMC8999052; doi:10.3390/ijms23073809)
Supplement: Supplementary file 1 [file ijms-23-03809-s001.zip › SUPPLEMENTARY FILES/S1_Lipid metabolism- and fibrosis-related genes.pdf]

Lipid Metabolism-Related genes

1) Fatty Acid Metabolism

a) Acetyl-CoA Transferases: ACAA1A, ACAA2, ACAT1, ACAT2

b) Acyl-CoA Dehydrogenases: ACAD10, ACAD11, ACAD9, ACADL, ACADM, ACADS, ACADSB, ACADVL, EHHADH, GCDH

c) Acyl-CoA Oxidases: ACOX1, ACOX2, ACOX3

d) Acyl-CoA Synthetases: ACSBG1, ACSBG2, ACSL1, ACSL3, ACSL4, ACSL5, ACSL6, ACSM2, ACSM3, ACSM4, ACSM5

e) Acyl-CoA Thioesterases: ACOT12, ACOT2, ACOT3, ACOT6, ACOT7, ACOT8, ACOT9

f) Carnitine Transferases: CPT1A, CPT1B, CPT1C, CPT2, CRAT, CROT

g) Other Fatty Acid Metabolism Genes: ALDH2, DECR1, DECR2, ECHS1, ECI2 (ACBD2, PECI), HADHA, MCEE, MUT, PECR, PPA1

2) Fatty Acid Transport

CPT1A, CPT1B, CPT1C, CPT2, CRAT, CROT, FABP1, FABP2, FABP3, FABP4, FABP5, FABP6, SLC27A1, SLC27A2, SLC27A3, SLC27A4, SLC27A5, SLC27A6

3) Fatty Acid Biosynthesis Regulation

PRKAA1 (AMPK), PRKAA2, PRKAB1, PRKAB2, PRKACA, PRKACB, PRKAG1, PRKAG2, PRKAG3

4) Ketogenesis & Ketone Body Metabolism

BDH1, BDH2, HMGCL, HMGCS1, HMGCS2, OXCT2A

5) Triacylglycerol Metabolism

GK, GK2, GPD1, GPD2, LIPE, LPL

Fibrosis-Related genes

1) Pro-Fibrotic

ACTA2 (A-SMA), AGT, CCL11 (EOTAXIN), CCL12 (MCP-5, SCYA12), CCL3 (MIP-1A), CCN2, GREM1, IL13, IL13RA2, IL4, IL5, SNAI1 (SNAIL)

2) Anti-Fibrotic

BMP7, HGF, IFNG, IL10, IL13RA2

3) Extracellular Matrix (ECM) & Cell Adhesion Molecules

a) Extracellular Matrix (ECM) Structural Constituents: COL1A2, COL3A1

b) Extracellular Matrix (ECM) Remodeling Enzymes: LOX, MMP13, MMP14, MMP1A, MMP2, MMP3, MMP8, MMP9, PLAT (TPA), PLAU (UPA), PLG, SERPINA1A, SERPINE1 (PAI-1), SERPINH1 (HSP47), TIMP1, TIMP2, TIMP3, TIMP4

c) Cell Adhesion Molecules: ITGA1, ITGA2, ITGA3, ITGAV, ITGB1, ITGB3, ITGB5, ITGB6, ITGB8

4) Inflammatory Cytokines & Chemokines

CCL11 (EOTAXIN), CCL12 (MCP-5, SCYA12), CCL3 (MIP-1A), CCR2, CXCR4, IFNG, IL10, IL13, IL13RA2, IL1A, IL1B, IL4, IL5, ILK, TNF

5) Growth Factors

AGT, CCN2, EDN1, EGF, HGF, PDGFA, PDGFB, VEGFA

6) Signal Transduction

a) TGFB Superfamily Members: BMP7, CAV1, DCN, ENG (EVI-1), GREM1, INHBE, LTBP1, SMAD2, SMAD3, SMAD4, SMAD6, SMAD7, TGFB1, TGFB2, TGFB3, TGFBR1 (ALK5), TGFBR2, TGIF1, THBS1 (TSP-1), THBS2

b) Transcription Factors: CEBPB, JUN, MYC, NFKB1, SP1, STAT1, STAT6

7) Epithelial-to-Mesenchymal Transition (EMT)

AKT1, BMP7, COL1A2, COL3A1, ITGAV, ITGB1, MMP2, MMP3, MMP9, SERPINE1 (PAI-1), SMAD2, SNAI1 (SNAIL), TGFB1, TGFB2, TGFB3, TIMP1

8) Other Fibrosis Genes

BCL2, FASL
