# Supplementary material for: A Wide-Proteome Analysis to Identify Molecular Pathways Involved in Kidney Response to High-Fat Diet in Mice
Source: Int J Mol Sci. 2022 Mar 30;23(7):3809. doi: 10.3390/ijms23073809 (PMC8999052; doi:10.3390/ijms23073809)
Supplement: Supplementary file 1 [file ijms-23-03809-s001.zip › SUPPLEMENTARY FILES/S7_Histological analyses.pdf]

Hystological anlayses

Table S1. General morphology.

	CORTEX			MEDULLA		INTERSTITIUM	VESSELS	PELVIS	MC	RC	PAS
**Case**	**Prox convoluted tubules**	**Distal conv. tubules**	**Glomeruli**	**Henle**	**Collectors**						
FAT 14	Tubular vacuolation (Lipidosis)	0	0	0	0	0	0	Mild lymphoplasmacytic inflammation	0	0	0
FAT 13	Tubular vacuolation (Lipidosis)	0	0	0	0	0	0	Minimal Mild lymphoplasmacytic inflammation	0	0	0
FAT 12	Tubular vacuolation (Lipidosis)	0	0	0	0	0	0	Minimal lymphoplasmacytic inflammation	0	0	0
FAT 11	Tubular vacuolation (Lipidosis)	0	0	0	0	0	0	Moderate lymphoplasmacytic inflammation	0	0	0
FAT 10	Tubular vacuolation (Lipidosis)	0	0	0	0	0	0	Minimal lymphoplasmacytic inflammation	0	0	0
LEAN 14	0	0	0	0	0	0	0	Minimal lymphoplasmacytic inflammation	0	0	0
LEAN 13	0	0	0	0	0	0	0	Minimal lymphoplasmacytic inflammation	1	0	0
LEAN 12	0	0	0	0	0	0	0	Minimal lymphoplasmacytic inflammation	1	0	0
LEAN 11	0	0	0	0	0	0	0	Minimal lymphoplasmacytic inflammation	0	0	0
LEAN 10	0	0	0	0	0	0	0	Minimal lymphoplasmacytic inflammation	0	0	0

Table S2. Lesional score (From Glastras et al 2016 modified for the area of assessment).

CASE	Fibrosis<br>Massons Thricrome/PAS<br>Lesional score as in M&M		Tubular lesions (PAS)<br>Lesional score as in M&M			
	Tubulointerstitial fibrosis	Glomerulosclerosis	Vacuolation	Dilation	Glycogenated nuclei	Casts
FAT 14	0	0	1	0	0	0
FAT 13	0	0	2	0	0	0
FAT 12	0	0	3	0	0	0
FAT 11	1	0	2	0	1	0
FAT 10	0	0	3	0	0	0
LEAN 14	0	0	0	0	0	0
LEAN 13	1	0	0	0	0	0
LEAN 12	1	0	0	0	0	0
LEAN 11	0	0	0	0	0	0
LEAN 10	0	0	0	0	0	0

Table S3. Glycogenated nuclear counts.

Case	400X magnification X ocular 10X/ FN20 (12 fields= standard area 2.37 mm^2)												Total count
	**1**	**2**	**3**	**4**	**5**	**6**	**7**	**8**	**9**	**10**	**11**	**12**	
FAT14	0	0	0	0	0	0	0	0	0	0	0	0	0
FAT13	0	1	0	0	0	0	0	0	0	0	0	1	2
FAT12	1	0	0	1	0	0	0	1	0	0	1	1	5
FAT11	0	0	0	1	0	3	2	2	0	2	1	0	9
FAT10	1	0	0	0	0	0	0	3	0	0	0	0	4
LEAN14	0	0	0	0	0	0	0	0	0	0	0	0	0
LEAN13	0	0	0	0	0	0	0	0	1	0	0	0	1
LEAN12	0	0	0	0	1	0	2	0	0	0	1	0	4
LEAN11	0	0	0	0	0	0	0	0	1	0	0	0	1
LEAN10	0	0	0	0	0	0	0	0	0	0	0	0	0
